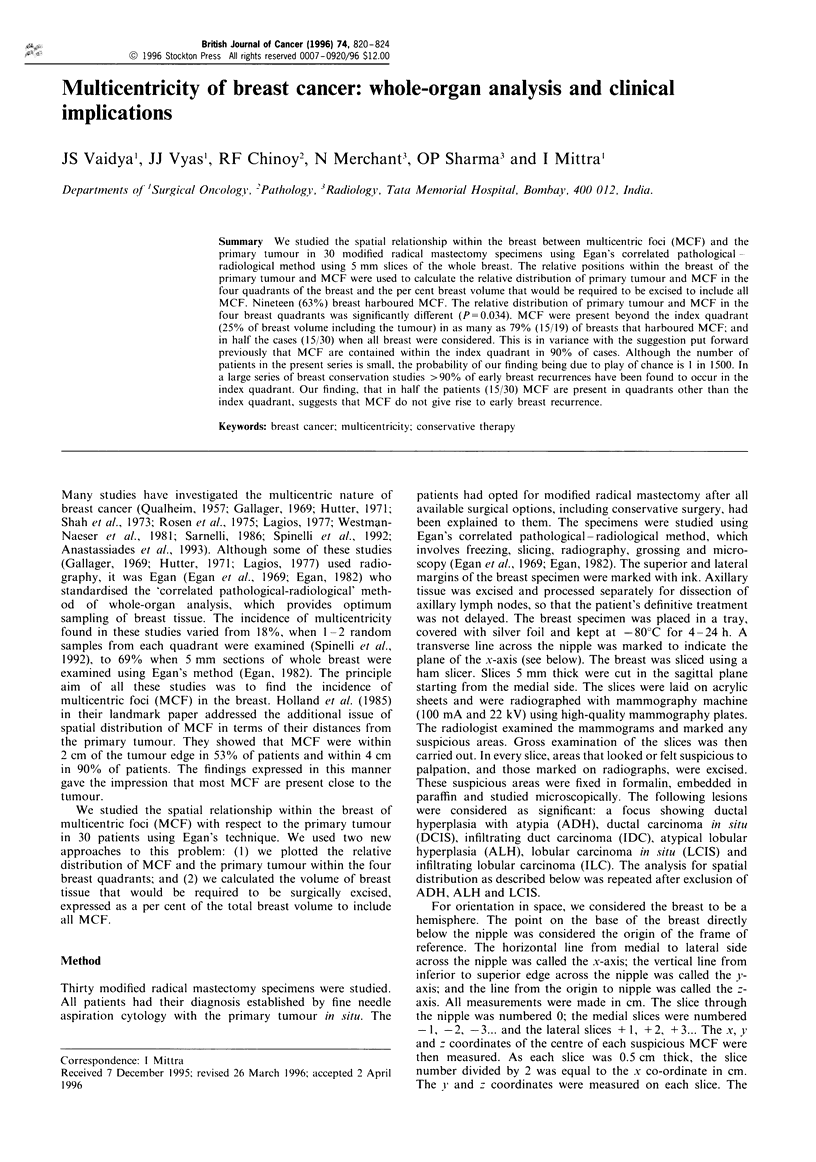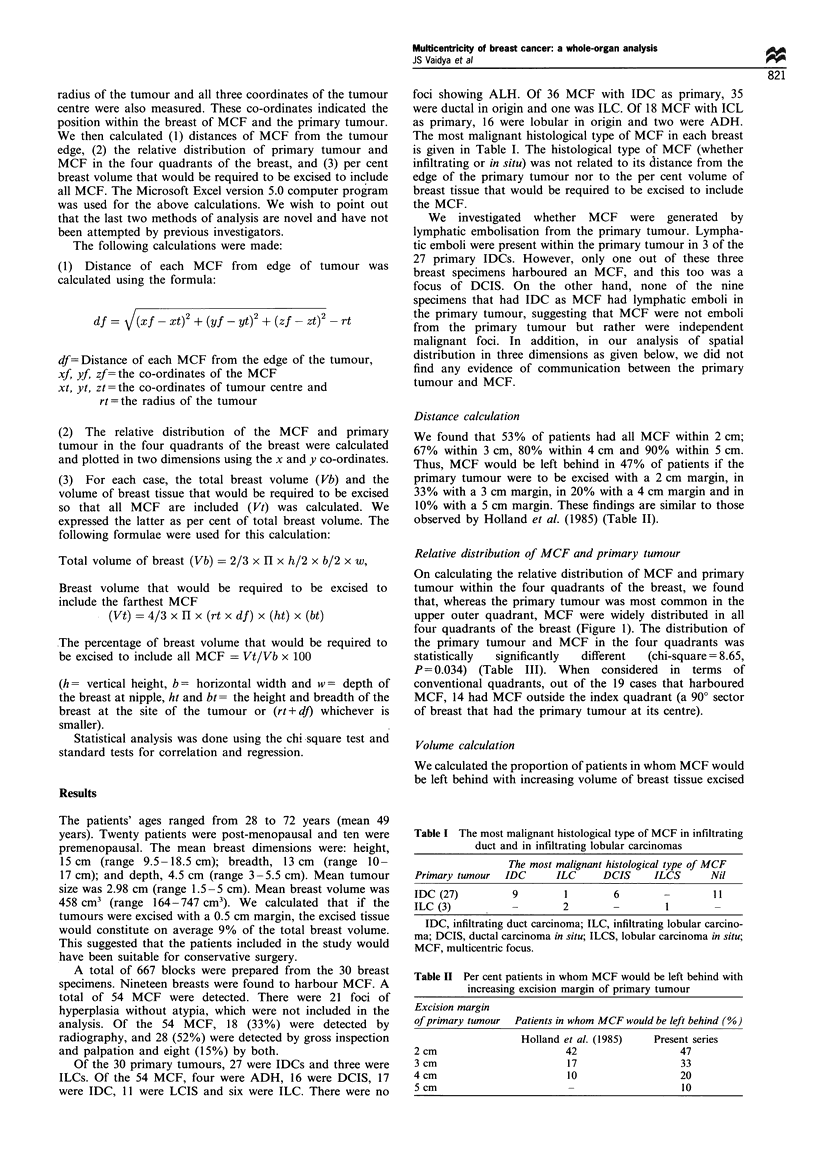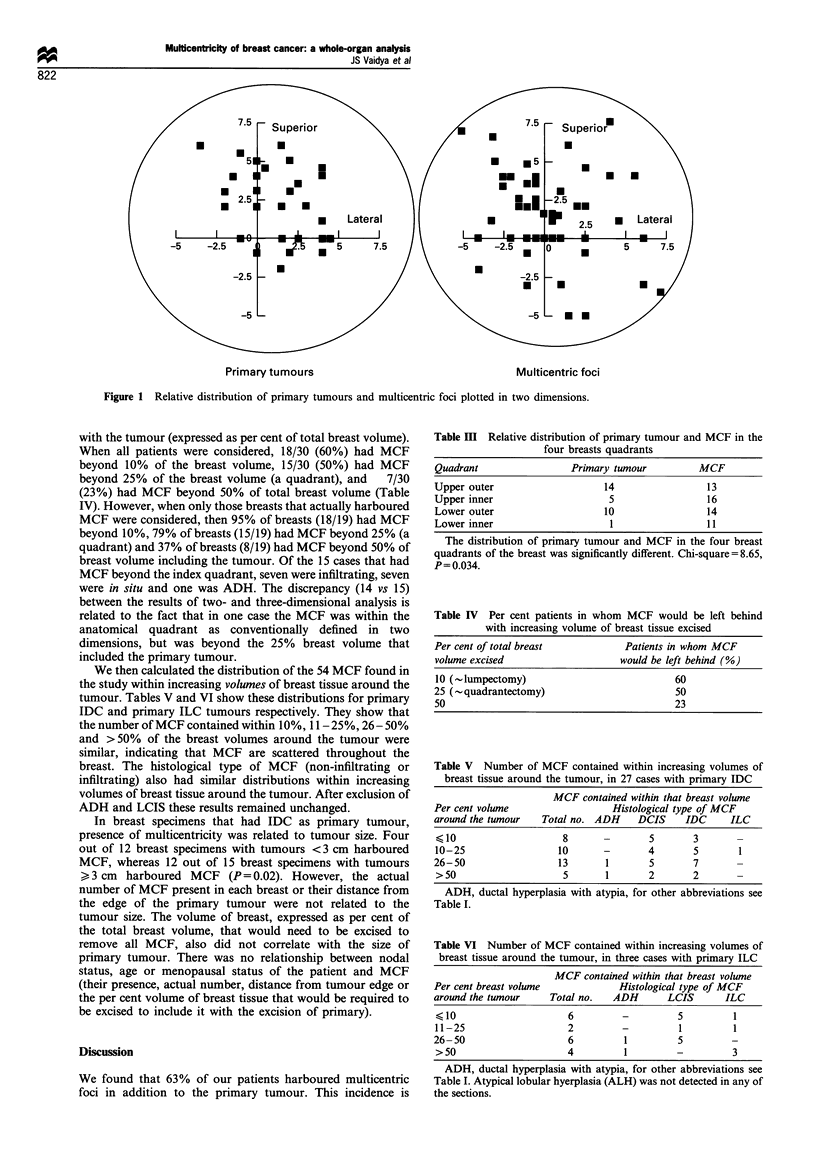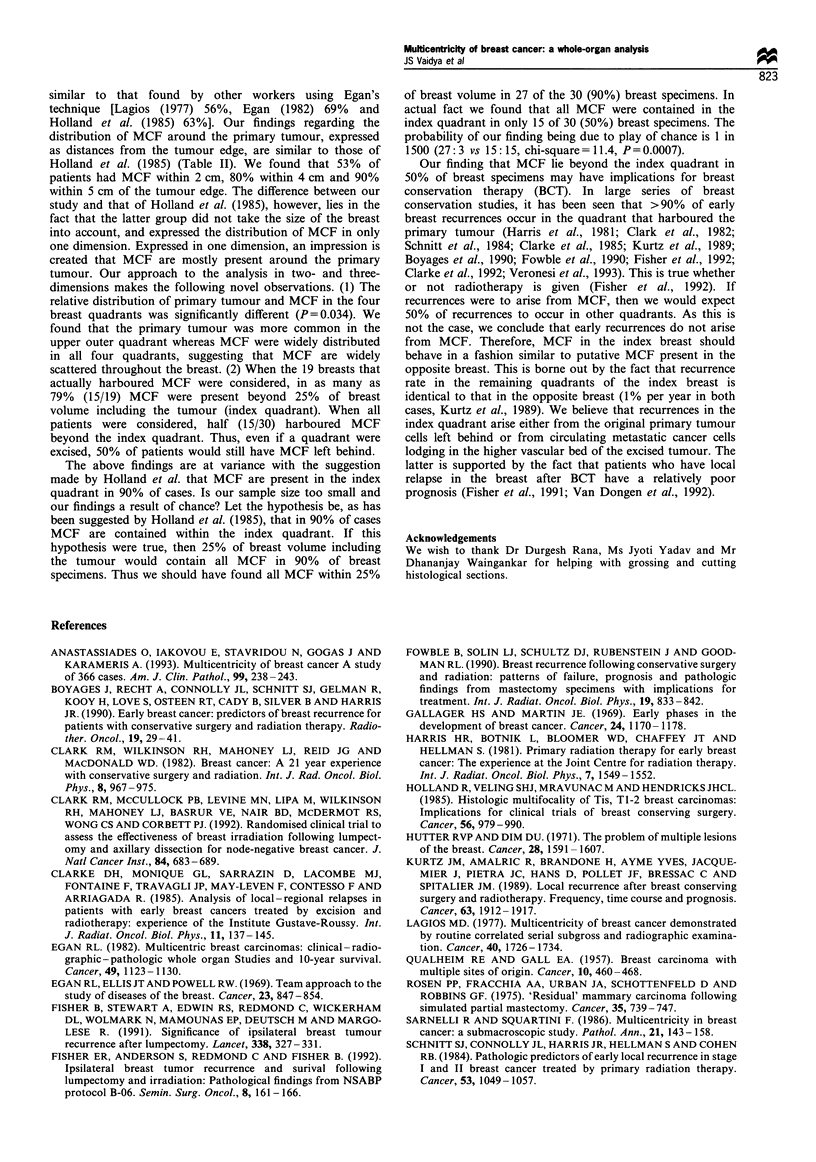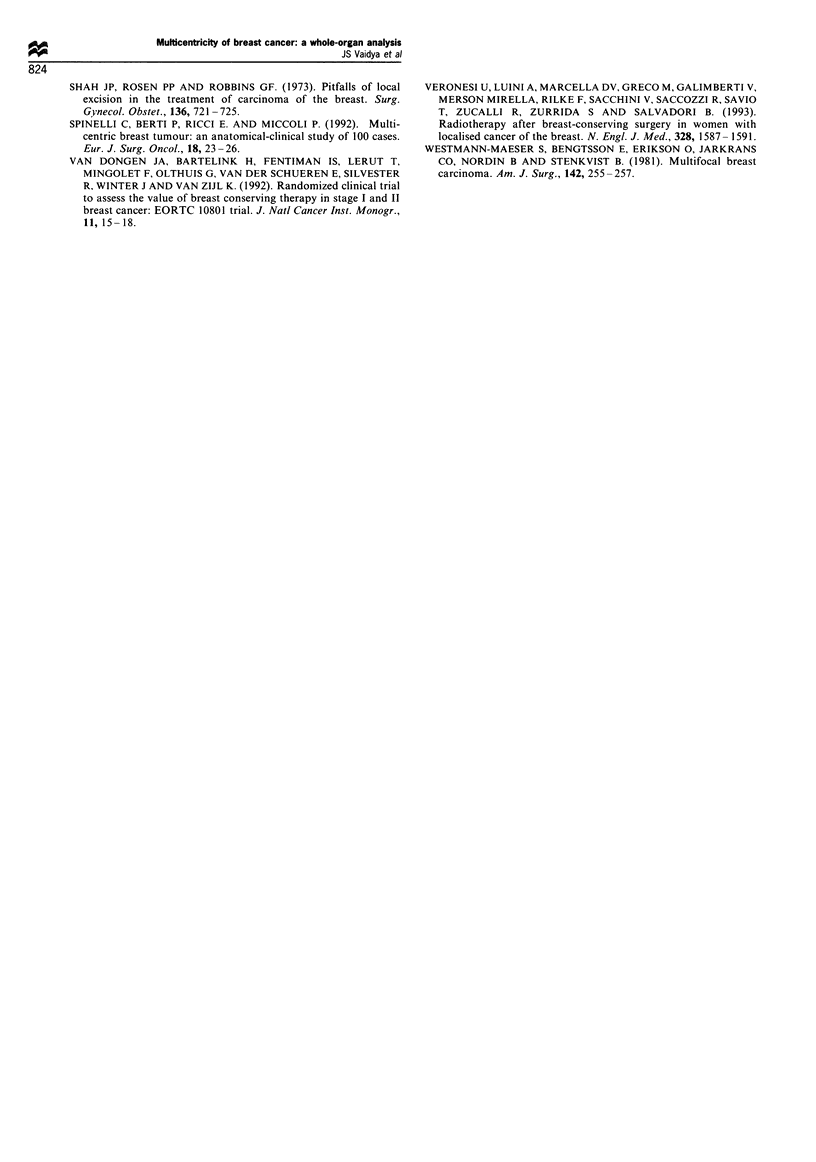# Multicentricity of breast cancer: whole-organ analysis and clinical implications.

**DOI:** 10.1038/bjc.1996.442

**Published:** 1996-09

**Authors:** J. S. Vaidya, J. J. Vyas, R. F. Chinoy, N. Merchant, O. P. Sharma, I. Mittra

**Affiliations:** Department of Surgical Oncology, Tata Memorial Hospital, Bombay, India.

## Abstract

We studied the spatial relationship within the breast between multicentric foci (MCF) and the primary tumour in 30 modified radical mastectomy specimens using Egan's correlated pathological-radiological method using 5 mm slices of the whole breast. The relative positions within the breast of the primary tumour and MCF were used to calculate the relative distribution of primary tumour and MCF in the four quadrants of the breast and the per cent breast volume that would be required to be excised to include all MCF. Nineteen (63%) breast harboured MCF. The relative distribution of primary tumour and MCF in the four breast quadrants was significantly different (P = 0.034). MCF were present beyond the index quadrant (25% of breast volume including the tumour) in as many as 79% (15/19) of breasts that harboured MCF; and in half the cases (15/30) when all breast were considered. This is in variance with the suggestion put forward previously that MCF are contained within the index quadrant in 90% of cases. Although the number of patients in the present series is small, the probability of our finding being due to play of chance is 1 in 1500. In a large series of breast conservation studies > 90% of early breast recurrences have been found to occur in the index quadrant. Our finding, that in half the patients (15/30) MCF are present in quadrants other than the index quadrant, suggests that MCF do not give rise to early breast recurrence.


British Journal of Cancer (1996) 74, 820-824
© 1996 Stockton Press All rights reserved 0007-0920/96 $12.00

### Multicentricity of breast cancer: whole-organ analysis and clinical implications

JS Vaidya[1], JJ Vyas[1], RF Chinoy[2], N Merchant[3], OP Sharma[3] and I Mittra[1]

*Departments of [1]Surgical Oncology, [2]Pathology, [3]Radiology, Tata Memorial Hospital, Bombay, 400 012, India.*

**Summary** We studied the spatial relationship within the breast between multicentric foci (MCF) and the primary tumour in 30 modified radical mastectomy specimens using Egan's correlated pathological-radiological method using 5 mm slices of the whole breast. The relative positions within the breast of the primary tumour and MCF were used to calculate the relative distribution of primary tumour and MCF in the four quadrants of the breast and the per cent breast volume that would be required to be excised to include all MCF. Nineteen (63%) breast harboured MCF. The relative distribution of primary tumour and MCF in the four breast quadrants was significantly different ($P=0.034$). MCF were present beyond the index quadrant (25% of breast volume including the tumour) in as many as 79% (15/19) of breasts that harboured MCF; and in half the cases (15/30) when all breast were considered. This is in variance with the suggestion put forward previously that MCF are contained within the index quadrant in 90% of cases. Although the number of patients in the present series is small, the probability of our finding being due to play of chance is 1 in 1500. In a large series of breast conservation studies >90% of early breast recurrences have been found to occur in the index quadrant. Our finding, that in half the patients (15/30) MCF are present in quadrants other than the index quadrant, suggests that MCF do not give rise to early breast recurrence.

**Keywords:** breast cancer; multicentricity; conservative therapy

Many studies have investigated the multicentric nature of breast cancer (Qualheim, 1957; Gallager, 1969; Hutter, 1971; Shah *et al.*, 1973; Rosen *et al.*, 1975; Lagios, 1977; Westman-Naeser *et al.*, 1981; Sarnelli, 1986; Spinelli *et al.*, 1992; Anastassiades *et al.*, 1993). Although some of these studies (Gallager, 1969; Hutter, 1971; Lagios, 1977) used radiography, it was Egan (Egan *et al.*, 1969; Egan, 1982) who standardised the 'correlated pathological-radiological' method of whole-organ analysis, which provides optimum sampling of breast tissue. The incidence of multicentricity found in these studies varied from 18%, when 1-2 random samples from each quadrant were examined (Spinelli *et al.*, 1992), to 69% when 5 mm sections of whole breast were examined using Egan's method (Egan, 1982). The principle aim of all these studies was to find the incidence of multicentric foci (MCF) in the breast. Holland *et al.* (1985) in their landmark paper addressed the additional issue of spatial distribution of MCF in terms of their distances from the primary tumour. They showed that MCF were within 2 cm of the tumour edge in 53% of patients and within 4 cm in 90% of patients. The findings expressed in this manner gave the impression that most MCF are present close to the tumour.

We studied the spatial relationship within the breast of multicentric foci (MCF) with respect to the primary tumour in 30 patients using Egan's technique. We used two new approaches to this problem: (1) we plotted the relative distribution of MCF and the primary tumour within the four breast quadrants; and (2) we calculated the volume of breast tissue that would be required to be surgically excised, expressed as a per cent of the total breast volume to include all MCF.

### Method

Thirty modified radical mastectomy specimens were studied. All patients had their diagnosis established by fine needle aspiration cytology with the primary tumour *in situ*. The patients had opted for modified radical mastectomy after all available surgical options, including conservative surgery, had been explained to them. The specimens were studied using Egan's correlated pathological-radiological method, which involves freezing, slicing, radiography, grossing and microscopy (Egan *et al.*, 1969; Egan, 1982). The superior and lateral margins of the breast specimen were marked with ink. Axillary tissue was excised and processed separately for dissection of axillary lymph nodes, so that the patient's definitive treatment was not delayed. The breast specimen was placed in a tray, covered with silver foil and kept at $-80°C$ for 4-24 h. A transverse line across the nipple was marked to indicate the plane of the $x$-axis (see below). The breast was sliced using a ham slicer. Slices 5 mm thick were cut in the sagittal plane starting from the medial side. The slices were laid on acrylic sheets and were radiographed with mammography machine (100 mA and 22 kV) using high-quality mammography plates. The radiologist examined the mammograms and marked any suspicious areas. Gross examination of the slices was then carried out. In every slice, areas that looked or felt suspicious to palpation, and those marked on radiographs, were excised. These suspicious areas were fixed in formalin, embedded in paraffin and studied microscopically. The following lesions were considered as significant: a focus showing ductal hyperplasia with atypia (ADH), ductal carcinoma *in situ* (DCIS), infiltrating duct carcinoma (IDC), atypical lobular hyperplasia (ALH), lobular carcinoma *in situ* (LCIS) and infiltrating lobular carcinoma (ILC). The analysis for spatial distribution as described below was repeated after exclusion of ADH, ALH and LCIS.

For orientation in space, we considered the breast to be a hemisphere. The point on the base of the breast directly below the nipple was considered the origin of the frame of reference. The horizontal line from medial to lateral side across the nipple was called the $x$-axis; the vertical line from inferior to superior edge across the nipple was called the $y$-axis; and the line from the origin to nipple was called the $z$-axis. All measurements were made in cm. The slice through the nipple was numbered 0; the medial slices were numbered $-1$, $-2$, $-3$... and the lateral slices $+1$, $+2$, $+3$... The $x$, $y$ and $z$ coordinates of the centre of each suspicious MCF were then measured. As each slice was 0.5 cm thick, the slice number divided by 2 was equal to the $x$ co-ordinate in cm. The $y$ and $z$ coordinates were measured on each slice. The

Correspondence: I Mittra
Received 7 December 1995; revised 26 March 1996; accepted 2 April 1996

radius of the tumour and all three coordinates of the tumour centre were also measured. These co-ordinates indicated the position within the breast of MCF and the primary tumour. We then calculated (1) distances of MCF from the tumour edge, (2) the relative distribution of primary tumour and MCF in the four quadrants of the breast, and (3) per cent breast volume that would be required to be excised to include all MCF. The Microsoft Excel version 5.0 computer program was used for the above calculations. We wish to point out that the last two methods of analysis are novel and have not been attempted by previous investigators.

The following calculations were made:

(1) Distance of each MCF from edge of tumour was calculated using the formula:

$$df = \sqrt{(xf - xt)^2 + (yf - yt)^2 + (zf - zt)^2} - rt$$

*df* = Distance of each MCF from the edge of the tumour,
*xf, yf, zf* = the co-ordinates of the MCF
*xt, yt, zt* = the co-ordinates of tumour centre and
*rt* = the radius of the tumour

(2) The relative distribution of the MCF and primary tumour in the four quadrants of the breast were calculated and plotted in two dimensions using the *x* and *y* co-ordinates.

(3) For each case, the total breast volume (*Vb*) and the volume of breast tissue that would be required to be excised so that all MCF are included (*Vt*) was calculated. We expressed the latter as per cent of total breast volume. The following formulae were used for this calculation:

Total volume of breast $(Vb) = 2/3 \times \Pi \times h/2 \times b/2 \times w$,

Breast volume that would be required to be excised to include the farthest MCF
$(Vt) = 4/3 \times \Pi \times (rt \times df) \times (ht) \times (bt)$

The percentage of breast volume that would be required to be excised to include all MCF $= Vt/Vb \times 100$

(*h* = vertical height, *b* = horizontal width and *w* = depth of the breast at nipple, *ht* and *bt* = the height and breadth of the breast at the site of the tumour or (*rt* + *df*) whichever is smaller).

Statistical analysis was done using the chi square test and standard tests for correlation and regression.

### Results

The patients' ages ranged from 28 to 72 years (mean 49 years). Twenty patients were post-menopausal and ten were premenopausal. The mean breast dimensions were: height, 15 cm (range 9.5–18.5 cm); breadth, 13 cm (range 10–17 cm); and depth, 4.5 cm (range 3–5.5 cm). Mean tumour size was 2.98 cm (range 1.5–5 cm). Mean breast volume was 458 $cm^3$ (range 164–747 $cm^3$). We calculated that if the tumours were excised with a 0.5 cm margin, the excised tissue would constitute on average 9% of the total breast volume. This suggested that the patients included in the study would have been suitable for conservative surgery.

A total of 667 blocks were prepared from the 30 breast specimens. Nineteen breasts were found to harbour MCF. A total of 54 MCF were detected. There were 21 foci of hyperplasia without atypia, which were not included in the analysis. Of the 54 MCF, 18 (33%) were detected by radiography, and 28 (52%) were detected by gross inspection and palpation and eight (15%) by both.

Of the 30 primary tumours, 27 were IDCs and three were ILCs. Of the 54 MCF, four were ADH, 16 were DCIS, 17 were IDC, 11 were LCIS and six were ILC. There were no foci showing ALH. Of 36 MCF with IDC as primary, 35 were ductal in origin and one was ILC. Of 18 MCF with ICL as primary, 16 were lobular in origin and two were ADH. The most malignant histological type of MCF in each breast is given in Table I. The histological type of MCF (whether infiltrating or *in situ*) was not related to its distance from the edge of the primary tumour nor to the per cent volume of breast tissue that would be required to be excised to include the MCF.

We investigated whether MCF were generated by lymphatic embolisation from the primary tumour. Lymphatic emboli were present within the primary tumour in 3 of the 27 primary IDCs. However, only one out of these three breast specimens harboured an MCF, and this too was a focus of DCIS. On the other hand, none of the nine specimens that had IDC as MCF had lymphatic emboli in the primary tumour, suggesting that MCF were not emboli from the primary tumour but rather were independent malignant foci. In addition, in our analysis of spatial distribution in three dimensions as given below, we did not find any evidence of communication between the primary tumour and MCF.

### *Distance calculation*

We found that 53% of patients had all MCF within 2 cm; 67% within 3 cm, 80% within 4 cm and 90% within 5 cm. Thus, MCF would be left behind in 47% of patients if the primary tumour were to be excised with a 2 cm margin, in 33% with a 3 cm margin, in 20% with a 4 cm margin and in 10% with a 5 cm margin. These findings are similar to those observed by Holland *et al.* (1985) (Table II).

### *Relative distribution of MCF and primary tumour*

On calculating the relative distribution of MCF and primary tumour within the four quadrants of the breast, we found that, whereas the primary tumour was most common in the upper outer quadrant, MCF were widely distributed in all four quadrants of the breast (Figure 1). The distribution of the primary tumour and MCF in the four quadrants was statistically significantly different (chi-square = 8.65, $P = 0.034$) (Table III). When considered in terms of conventional quadrants, out of the 19 cases that harboured MCF, 14 had MCF outside the index quadrant (a 90° sector of breast that had the primary tumour at its centre).

### *Volume calculation*

We calculated the proportion of patients in whom MCF would be left behind with increasing volume of breast tissue excised

**Table I** The most malignant histological type of MCF in infiltrating duct and in infiltrating lobular carcinomas

| | *The most malignant histological type of MCF* | | | | |
|---|---|---|---|---|---|
| *Primary tumour* | *IDC* | *ILC* | *DCIS* | *ILCS* | *Nil* |
| IDC (27) | 9 | 1 | 6 | – | 11 |
| ILC (3) | – | 2 | – | 1 | – |

IDC, infiltrating duct carcinoma; ILC, infiltrating lobular carcinoma; DCIS, ductal carcinoma *in situ*; ILCS, lobular carcinoma *in situ*; MCF, multicentric focus.

**Table II** Per cent patients in whom MCF would be left behind with increasing excision margin of primary tumour

| *Excision margin of primary tumour* | *Patients in whom MCF would be left behind (%)* | |
|---|---|---|
| | Holland *et al.* (1985) | Present series |
| 2 cm | 42 | 47 |
| 3 cm | 17 | 33 |
| 4 cm | 10 | 20 |
| 5 cm | – | 10 |

**Figure 1** Relative distribution of primary tumours and multicentric foci plotted in two dimensions.

with the tumour (expressed as per cent of total breast volume). When all patients were considered, 18/30 (60%) had MCF beyond 10% of the breast volume, 15/30 (50%) had MCF beyond 25% of the breast volume (a quadrant), and 7/30 (23%) had MCF beyond 50% of total breast volume (Table IV). However, when only those breasts that actually harboured MCF were considered, then 95% of breasts (18/19) had MCF beyond 10%, 79% of breasts (15/19) had MCF beyond 25% (a quadrant) and 37% of breasts (8/19) had MCF beyond 50% of breast volume including the tumour. Of the 15 cases that had MCF beyond the index quadrant, seven were infiltrating, seven were *in situ* and one was ADH. The discrepancy (14 *vs* 15) between the results of two- and three-dimensional analysis is related to the fact that in one case the MCF was within the anatomical quadrant as conventionally defined in two dimensions, but was beyond the 25% breast volume that included the primary tumour.

We then calculated the distribution of the 54 MCF found in the study within increasing *volumes* of breast tissue around the tumour. Tables V and VI show these distributions for primary IDC and primary ILC tumours respectively. They show that the number of MCF contained within 10%, 11–25%, 26–50% and >50% of the breast volumes around the tumour were similar, indicating that MCF are scattered throughout the breast. The histological type of MCF (non-infiltrating or infiltrating) also had similar distributions within increasing volumes of breast tissue around the tumour. After exclusion of ADH and LCIS these results remained unchanged.

In breast specimens that had IDC as primary tumour, presence of multicentricity was related to tumour size. Four out of 12 breast specimens with tumours <3 cm harboured MCF, whereas 12 out of 15 breast specimens with tumours ⩾3 cm harboured MCF ($P$=0.02). However, the actual number of MCF present in each breast or their distance from the edge of the primary tumour were not related to the tumour size. The volume of breast, expressed as per cent of the total breast volume, that would need to be excised to remove all MCF, also did not correlate with the size of primary tumour. There was no relationship between nodal status, age or menopausal status of the patient and MCF (their presence, actual number, distance from tumour edge or the per cent volume of breast tissue that would be required to be excised to include it with the excision of primary).

### Discussion

We found that 63% of our patients harboured multicentric foci in addition to the primary tumour. This incidence is

**Table III** Relative distribution of primary tumour and MCF in the four breasts quadrants

| *Quadrant* | *Primary tumour* | *MCF* |
|---|---|---|
| Upper outer | 14 | 13 |
| Upper inner | 5 | 16 |
| Lower outer | 10 | 14 |
| Lower inner | 1 | 11 |

The distribution of primary tumour and MCF in the four breast quadrants of the breast was significantly different. Chi-square=8.65, $P$=0.034.

**Table IV** Per cent patients in whom MCF would be left behind with increasing volume of breast tissue excised

| *Per cent of total breast volume excised* | *Patients in whom MCF would be left behind (%)* |
|---|---|
| 10 (~lumpectomy) | 60 |
| 25 (~quadrantectomy) | 50 |
| 50 | 23 |

**Table V** Number of MCF contained within increasing volumes of breast tissue around the tumour, in 27 cases with primary IDC

| *Per cent volume around the tumour* | *MCF contained within that breast volume* *Total no.* | *Histological type of MCF* *ADH* | *DCIS* | *IDC* | *ILC* |
|---|---|---|---|---|---|
| ⩽10 | 8 | – | 5 | 3 | – |
| 10–25 | 10 | – | 4 | 5 | 1 |
| 26–50 | 13 | 1 | 5 | 7 | – |
| >50 | 5 | 1 | 2 | 2 | – |

ADH, ductal hyperplasia with atypia, for other abbreviations see Table I.

**Table VI** Number of MCF contained within increasing volumes of breast tissue around the tumour, in three cases with primary ILC

| *Per cent breast volume around the tumour* | *MCF contained within that breast volume* *Total no.* | *Histological type of MCF* *ADH* | *LCIS* | *ILC* |
|---|---|---|---|---|
| ⩽10 | 6 | – | 5 | 1 |
| 11–25 | 2 | – | 1 | 1 |
| 26–50 | 6 | 1 | 5 | – |
| >50 | 4 | 1 | – | 3 |

ADH, ductal hyperplasia with atypia, for other abbreviations see Table I. Atypical lobular hyerplasia (ALH) was not detected in any of the sections.

similar to that found by other workers using Egan's technique [Lagios (1977) 56%, Egan (1982) 69% and Holland *et al.* (1985) 63%]. Our findings regarding the distribution of MCF around the primary tumour, expressed as distances from the tumour edge, are similar to those of Holland *et al.* (1985) (Table II). We found that 53% of patients had MCF within 2 cm, 80% within 4 cm and 90% within 5 cm of the tumour edge. The difference between our study and that of Holland *et al.* (1985), however, lies in the fact that the latter group did not take the size of the breast into account, and expressed the distribution of MCF in only one dimension. Expressed in one dimension, an impression is created that MCF are mostly present around the primary tumour. Our approach to the analysis in two- and three-dimensions makes the following novel observations. (1) The relative distribution of primary tumour and MCF in the four breast quadrants was significantly different ($P = 0.034$). We found that the primary tumour was more common in the upper outer quadrant whereas MCF were widely distributed in all four quadrants, suggesting that MCF are widely scattered throughout the breast. (2) When the 19 breasts that actually harboured MCF were considered, in as many as 79% (15/19) MCF were present beyond 25% of breast volume including the tumour (index quadrant). When all patients were considered, half (15/30) harboured MCF beyond the index quadrant. Thus, even if a quadrant were excised, 50% of patients would still have MCF left behind.

The above findings are at variance with the suggestion made by Holland *et al.* that MCF are present in the index quadrant in 90% of cases. Is our sample size too small and our findings a result of chance? Let the hypothesis be, as has been suggested by Holland *et al.* (1985), that in 90% of cases MCF are contained within the index quadrant. If this hypothesis were true, then 25% of breast volume including the tumour would contain all MCF in 90% of breast specimens. Thus we should have found all MCF within 25% of breast volume in 27 of the 30 (90%) breast specimens. In actual fact we found that all MCF were contained in the index quadrant in only 15 of 30 (50%) breast specimens. The probability of our finding being due to play of chance is 1 in 1500 (27 : 3 *vs* 15 : 15, chi-square = 11.4, $P = 0.0007$).

Our finding that MCF lie beyond the index quadrant in 50% of breast specimens may have implications for breast conservation therapy (BCT). In large series of breast conservation studies, it has been seen that >90% of early breast recurrences occur in the quadrant that harboured the primary tumour (Harris *et al.*, 1981; Clark *et al.*, 1982; Schnitt *et al.*, 1984; Clarke *et al.*, 1985; Kurtz *et al.*, 1989; Boyages *et al.*, 1990; Fowble *et al.*, 1990; Fisher *et al.*, 1992; Clarke *et al.*, 1992; Veronesi *et al.*, 1993). This is true whether or not radiotherapy is given (Fisher *et al.*, 1992). If recurrences were to arise from MCF, then we would expect 50% of recurrences to occur in other quadrants. As this is not the case, we conclude that early recurrences do not arise from MCF. Therefore, MCF in the index breast should behave in a fashion similar to putative MCF present in the opposite breast. This is borne out by the fact that recurrence rate in the remaining quadrants of the index breast is identical to that in the opposite breast (1% per year in both cases, Kurtz *et al.*, 1989). We believe that recurrences in the index quadrant arise either from the original primary tumour cells left behind or from circulating metastatic cancer cells lodging in the higher vascular bed of the excised tumour. The latter is supported by the fact that patients who have local relapse in the breast after BCT have a relatively poor prognosis (Fisher *et al.*, 1991; Van Dongen *et al.*, 1992).

**Acknowledgements**
We wish to thank Dr Durgesh Rana, Ms Jyoti Yadav and Mr Dhananjay Waingankar for helping with grossing and cutting histological sections.

### References

ANASTASSIADES O, IAKOVOU E, STAVRIDOU N, GOGAS J AND KARAMERIS A. (1993). Multicentricity of breast cancer A study of 366 cases. *Am. J. Clin. Pathol.*, **99**, 238–243.

BOYAGES J, RECHT A, CONNOLLY JL, SCHNITT SJ, GELMAN R, KOOY H, LOVE S, OSTEEN RT, CADY B, SILVER B AND HARRIS JR. (1990). Early breast cancer: predictors of breast recurrence for patients with conservative surgery and radiation therapy. *Radiother. Oncol.*, **19**, 29–41.

CLARK RM, WILKINSON RH, MAHONEY LJ, REID JG AND MACDONALD WD. (1982). Breast cancer: A 21 year experience with conservative surgery and radiation. *Int. J. Rad. Oncol. Biol. Phys.*, **8**, 967–975.

CLARK RM, MCCULLOCK PB, LEVINE MN, LIPA M, WILKINSON RH, MAHONEY LJ, BASRUR VE, NAIR BD, MCDERMOT RS, WONG CS AND CORBETT PJ. (1992). Randomised clinical trial to assess the effectiveness of breast irradiation following lumpectomy and axillary dissection for node-negative breast cancer. *J. Natl Cancer Inst.*, **84**, 683–689.

CLARKE DH, MONIQUE GL, SARRAZIN D, LACOMBE MJ, FONTAINE F, TRAVAGLI JP, MAY-LEVEN F, CONTESSO F AND ARRIAGADA R. (1985). Analysis of local–regional relapses in patients with early breast cancers treated by excision and radiotherapy: experience of the Institute Gustave-Roussy. *Int. J. Radiat. Oncol. Biol. Phys.*, **11**, 137–145.

EGAN RL. (1982). Multicentric breast carcinomas: clinical–radiographic–pathologic whole organ Studies and 10-year survival. *Cancer*, **49**, 1123–1130.

EGAN RL, ELLIS JT AND POWELL RW. (1969). Team approach to the study of diseases of the breast. *Cancer*, **23**, 847–854.

FISHER B, STEWART A, EDWIN RS, REDMOND C, WICKERHAM DL, WOLMARK N, MAMOUNAS EP, DEUTSCH M AND MARGOLESE R. (1991). Significance of ipsilateral breast tumour recurrence after lumpectomy. *Lancet*, **338**, 327–331.

FISHER ER, ANDERSON S, REDMOND C AND FISHER B. (1992). Ipsilateral breast tumor recurrence and surival following lumpectomy and irradiation: Pathological findings from NSABP protocol B-06. *Semin. Surg. Oncol.*, **8**, 161–166.

FOWBLE B, SOLIN LJ, SCHULTZ DJ, RUBENSTEIN J AND GOODMAN RL. (1990). Breast recurrence following conservative surgery and radiation: patterns of failure, prognosis and pathologic findings from mastectomy specimens with implications for treatment. *Int. J. Radiat. Oncol. Biol. Phys.*, **19**, 833–842.

GALLAGER HS AND MARTIN JE. (1969). Early phases in the development of breast cancer. *Cancer*, **24**, 1170–1178.

HARRIS HR, BOTNIK L, BLOOMER WD, CHAFFEY JT AND HELLMAN S. (1981). Primary radiation therapy for early breast cancer: The experience at the Joint Centre for radiation therapy. *Int. J. Radiat. Oncol. Biol. Phys.*, **7**, 1549–1552.

HOLLAND R, VELING SHJ, MRAVUNAC M AND HENDRICKS JHCL. (1985). Histologic multifocality of Tis, T1-2 breast carcinomas: Implications for clinical trials of breast conserving surgery. *Cancer*, **56**, 979–990.

HUTTER RVP AND DIM DU. (1971). The problem of multiple lesions of the breast. *Cancer*, **28**, 1591–1607.

KURTZ JM, AMALRIC R, BRANDONE H, AYME YVES, JACQUEMIER J, PIETRA JC, HANS D, POLLET JF, BRESSAC C AND SPITALIER JM. (1989). Local recurrence after breast conserving surgery and radiotherapy. Frequency, time course and prognosis. *Cancer*, **63**, 1912–1917.

LAGIOS MD. (1977). Multicentricity of breast cancer demonstrated by routine correlated serial subgross and radiographic examination. *Cancer*, **40**, 1726–1734.

QUALHEIM RE AND GALL EA. (1957). Breast carcinoma with multiple sites of origin. *Cancer*, **10**, 460–468.

ROSEN PP, FRACCHIA AA, URBAN JA, SCHOTTENFELD D AND ROBBINS GF. (1975). 'Residual' mammary carcinoma following simulated partial mastectomy. *Cancer*, **35**, 739–747.

SARNELLI R AND SQUARTINI F. (1986). Multicentricity in breast cancer: a submacroscopic study. *Pathol. Ann.*, **21**, 143–158.

SCHNITT SJ, CONNOLLY JL, HARRIS JR, HELLMAN S AND COHEN RB. (1984). Pathologic predictors of early local recurrence in stage I and II breast cancer treated by primary radiation therapy. *Cancer*, **53**, 1049–1057.

SHAH JP, ROSEN PP AND ROBBINS GF. (1973). Pitfalls of local excision in the treatment of carcinoma of the breast. *Surg. Gynecol. Obstet.*, **136,** 721-725.

SPINELLI C, BERTI P, RICCI E. AND MICCOLI P. (1992). Multicentric breast tumour: an anatomical-clinical study of 100 cases. *Eur. J. Surg. Oncol.*, **18,** 23-26.

VAN DONGEN JA, BARTELINK H, FENTIMAN IS, LERUT T, MINGOLET F, OLTHUIS G, VAN DER SCHUEREN E, SILVESTER R, WINTER J AND VAN ZIJL K. (1992). Randomized clinical trial to assess the value of breast conserving therapy in stage I and II breast cancer: EORTC 10801 trial. *J. Natl Cancer Inst. Monogr.*, **11,** 15-18.

VERONESI U, LUINI A, MARCELLA DV, GRECO M, GALIMBERTI V, MERSON MIRELLA, RILKE F, SACCHINI V, SACCOZZI R, SAVIO T, ZUCALLI R, ZURRIDA S AND SALVADORI B. (1993). Radiotherapy after breast-conserving surgery in women with localised cancer of the breast. *N. Engl. J. Med.*, **328,** 1587-1591.

WESTMANN-MAESER S, BENGTSSON E, ERIKSON O, JARKRANS CO, NORDIN B AND STENKVIST B. (1981). Multifocal breast carcinoma. *Am. J. Surg.*, **142,** 255-257.